# Reassortment and genomic analysis of a G9P[8]-E2 rotavirus isolated in China

**DOI:** 10.1186/s12985-023-02064-5

**Published:** 2023-06-22

**Authors:** Rui Peng, Dandi Li, Jindong Wang, Guangping Xiong, Mengxuan Wang, Dan Liu, Yuhang Wei, Lili Pang, Xiaoman Sun, Huiying Li, Xiangyu Kong, Saleha Shahar, Zhaojun Duan

**Affiliations:** 1 Department of Biosciences, Faculty of Sciences, Universiti Teknologi Malaysia, Johor Bahru, 81310 Malaysia; 2 NHC Key Laboratory of Medical Viruses and Viral Diseases, Institute of Viral Disease Prevention and Control, National Health Commission, Chinese Centre for Disease Control and Prevention, Beijing, 102206 China; 3 College of Life Science, Hengshui University, Hengshui, 053000 China; 4 Department of Pathogenic Biology, Weifang Medical University, Weifang, 261053 China; 5 College of Public Health, Gansu University of Chinese Medicine, Lanzhou, 730000 China

**Keywords:** Evolution, G9P[8]-E2, Isolation, Neutralizing antigen epitope, Reassortment, Rotavirus

## Abstract

**Objective:**

To isolate a prevalent G9P[8] group A rotavirus (RVA) (N4006) in China and investigate its genomic and evolutionary characteristics, with the goal of facilitating the development of a new rotavirus vaccine.

**Methods:**

The RVA G9P[8] genotype from a diarrhea sample was passaged in MA104 cells. The virus was evaluated by TEM, polyacrylamide gel electrophoresis, and indirect immunofluorescence assay. The complete genome of virus was obtained by RT-PCR and sequencing. The genomic and evolutionary characteristics of the virus were evaluated by nucleic acid sequence analysis with MEGA ver. 5.0.5 and DNASTAR software. The neutralizing epitopes of VP7 and VP4 (VP5* and VP8*) were analyzed using BioEdit ver. 7.0.9.0 and PyMOL ver. 2.5.2.

**Results:**

The RVA N4006 (G9P[8] genotype) was adapted in MA104 cells with a high titer (10^5.5^ PFU/mL). Whole-genome sequence analysis showed N4006 to be a reassortant rotavirus of Wa-like G9P[8] RVA and the NSP4 gene of DS-1-like G2P[4] RVA, with the genotype constellation G9-P[8]-I1-R1-C1-M1-A1-N1-T1-E2-H1 (G9P[8]-E2). Phylogenetic analysis indicated that N4006 had a common ancestor with Japanese G9P[8]-E2 rotavirus. Neutralizing epitope analysis showed that VP7, VP5*, and VP8* of N4006 had low homology with vaccine viruses of the same genotype and marked differences with vaccine viruses of other genotypes.

**Conclusion:**

The RVA G9P[8] genotype with the G9-P[8]-I1-R1-C1-M1-A1-N1-T1-E2-H1 (G9P[8]-E2) constellation predominates in China and may originate from reassortment between Japanese G9P[8] with Japanese DS-1-like G2P[4] rotaviruses. The antigenic variation of N4006 with the vaccine virus necessitates an evaluation of the effect of the rotavirus vaccine on G9P[8]-E2 genotype rotavirus.

**Supplementary Information:**

The online version contains supplementary material available at 10.1186/s12985-023-02064-5.

## Background

Group A rotavirus is a major cause of diarrhea in infants and children < 5 years old. One research on the global burden and trends of rotavirus infection-related deaths from 1990 to 2019 showed that the trend of global rotavirus burden has significantly decreased over the past thirty years [1]. However, RVA-related annual mortality is still high. About 213,000 deaths in 2013 [2] and128,500 deaths in 2016 [3] were caused, and the deaths caused by rotavirus accounted for 19.11% of diarrhoea deaths in 2019 in the world [1]. RVA vaccines can prevent and control RVA-induced diarrhea and are in use worldwide.

The recombinant RotaTeq (RV5, Merck, Rahway, NJ, USA) and the Lanzhou lamb rotavirus vaccine (LLR; Lanzhou Institute of Biological Products, China) are the only two vaccines available in China. The RV5 vaccine is an attenuated reconstituted of a pentavalent human–bovine virus, which is recombined *in vitro* with WinStar calf 3 (WC3; G6P[8]) and five human epidemic viruses: G1P[8], G2P[4], G3P[8], G4P[8], and G9P[8] [3, 4]. The protective effectiveness of RV5 against all types of severe rotavirus gastroenteritis (RVGE) was 78.9% (95% confidence interval [CI]) according to phase III clinical trial data in China, and its protective effect against all levels of RVGE was 69.9% (95% CI). The protective efficacy against severe RVGE and other severities of RVGE caused by the G9 genotype was 88.3% (95% CI) and 67.4% (95% CI), respectively [5]. The lamb attenuated rotavirus with the G10P[15] genotype is the basis of the LLR vaccine. A post-marketing case–control study in Guangzhou, Zhengding, and Beijing, China, revealed that LLR had a protective efficacy of 52–88% against severe RVGE and 35% against RVGE of any severity. The vaccines had a heterotypic protective effect against severe RVGE caused by G3 and G9 of 52% and 40%, respectively [6].

The parent viruses of current RVA vaccines were developed from prevalent viruses 20 or 30 years ago. For example, the parental RVA strains of RotaTeq with human G1–G4 and P[8] genotypes and the orally attenuated live vaccine ROTARIX (RV1; GSK, Brentford, UK) with human G1P[8] genotypes were isolated more than 30 years ago [7]. Genetic mutation, recombination, and reassortment continuously alter the nucleotide sequences and genotypes of common RVA, potentially affecting the protective efficacies of vaccines.

RVAs can be classified into Wa-like (I1-R1-C1-M1-1-A1-1-N1-T1-T 1-E1-H1), DS-1-like (I2-R2-C2-M2-M 2-A2-N2-N 2-T2-E2-H2), and AU-1-like (I3-R3-C3-M3-A3/A12-N3-T3-E3-H3/H6). Currently, most RVAs are Wa-like [8–10]. Among RVAs, G1P[8], G3[8], and G9[8] genotypes are typically Wa-like, whereas G2P[4] and G8P[8] are usually DS-1-like [11]. In China, the major epidemic viruses from 1998 to 2000 were of G1P[8] genotype (72.7%). From August 2003 to July 2007, viruses of the G3 genotype predominated (> 50%). The proportion of G9P[8] RVA increased rapidly from 3.4% to 2009 to 91.56% in 2018 [12–15]. Genome-wide sequence analysis was performed on 64 samples of G9P[8] genotype RVA in Shenzhen and Changchun, China, in 2018. Among the 54 viruses (30 in Shenzhen and 24 in Changchun) whose genome-wide fragments were amplified, 27 were Wa-like G9P[8]-E1 with a G9-P[8]-I1-R1-C1-M1-A1-N1-T1-E1-H1 genetic constellation, and 27 were Wa-like G9P[8]-E2 with a G9-P[8]-I1-R1-C1-M1-A1-N1-T1-E2-H1 genetic constellation. The RVA G9[8]-E2 accounted for 60% and 33% of the totals in Shenzhen and Changchun, respectively [16]. According to surveillance data of the Chinese Center for Disease Control and Prevention (China CDC), G9P[8]-E2 has spread widely in China (data not shown).

The protective effect of RVA vaccines is mediated by a homotypic immune response against the same G/P genotype of the vaccine, whereas the heterotypic or cross-reaction response is poor [17]. The LLR virus in China does not belong to any of the prevalent genotypes, and its reported protective effect is low. Although RV5 covers previously epidemic genotypes, sequence changes in the prevalent genotype might have reduced its immunogenicity and protective effect. It is important to isolate G9P[8] RVA, the most common virus in China, for vaccine evaluation and development. In this study, a G9P[8] RVA (N4006) was isolated from a child with diarrhea in China. We investigated its evolutionary, genetic, and antigenic characteristics.

## Methods

### Sample and MA104 cells

The diarrhea sample N4006 was collected from a child aged 27 months with diarrhea hospitalized at the Children’s Hospital Capital Institute of Pediatrics (Beijing, China) in 2020. The sample contained only RVA G9P[8] but without other common children’s enteric viruses like calicivirus, adenovirus, and astrovirus through PCR and rotavirus G/P genotyping [18]. An African Green Monkey fetal kidney epithelial cell line (MA104) used in this study was obtained from the Cell Culture Laboratory, NHC Key Laboratory of Medical Viruses and Viral Diseases, China CDC, Beijing, China.

### Fecal sample preparation

Fecal sample was prepared to achieve a 10% stool suspension. Specifically, the stool sample and phosphate-buffered saline were added into a 1.5ml Eppendorf tube and shaken for 30 s. The mixture was left to stand for 10 min at room temperature, and then centrifuged at 5000 ×g for 5 min. Finally, the supernatant was collected for the subsequent tests.

### RVA isolation

The RVA in this sample (N4006) was isolated by passaging 10 times and plaque-purifying twice in MA104 cells. RVA passage culture was performed as follows. MA104 cells were seeded into roller tubes and cultured in Dulbecco’s modified Eagle’s medium (DMEM) containing 10% fetal bovine serum. When the cells reached 90–100% confluence, 0.05% trypsin-EDTA (25300-054, Gibco, Canada) was used for disruption and the cells were inoculated into roller tubes in an incubator with a 5% CO_2_ atmosphere at 37℃. The MA104 cells formed monolayers in 2 days. Next, RVA was activated with 800 µg/mL CaCl_2_ and 15 µg/mL trypsin (15050-065, Gibco, NY_14072, USA) at 37℃ for 1 h. Subsequently, MA104 cells in roller tubes were washed with serum-free DMEM to remove the remaining serum, added to activated RVA, and placed in a CO_2_ incubator for 2 h for virus adsorption. Next, the MA104 cells were added to DMEM containing 20 µg/mL trypsin (15050-065, Gibco, NY_14072, USA) to culture RVA. Cells without cytopathic effect (CPE) were harvested on the fifth day and were subjected to subculture until the CPE occurred. The culture was freeze-thawed thrice and harvested by centrifugation at 2000 ×g for 5 min [19]. RVA passaging was conducted under good laboratory practice conditions using previously reported methods and certified reagents.

### Plaque purifying

Trypsin was added to different dilutions (10^− 1^,10^− 2^,10^–3^, 10^− 4^ and 10^− 5^) of rotavirus solutions to the final concentration of 15 µg/ml. After incubating in a 37 ℃ water bath for 1 h, the virus solutions were added into the monolayer MA104 cells. After 2 hours’ of virus adsorption process in a 37 ℃ water bath, the MA104 cells were covered with 1.6% low melting temperature agarose. Virus-free MA104 cells covered with the same low melting temperature agarose were set as a negative control. Plaque formation was observed daily and picked out for culturing in MA104 cells so as to obtain purified rotavirus.

### RVA identification and growth curve determination

The isolated RVA was identified by transmission electron microscopy (TEM) and polyacrylamide gel electrophoresis (PAGE). For TEM, in brief, after centrifuging the freeze-thawed culture for 30 min at 8000 ×g, the supernatant was centrifuged at 100,000 ×g for 2 h to concentrate the virus. Subsequently, the virus was observed under the TEM after sediment resuspension with phosphate buffered saline and negative staining with phosphor tungstic acid. For PAGE, genomic RNA of the 5th, 7th and 10th generation rotaviruses and that of SA11 (G3P[3]) and RotaTeq W179-9 (G1P[5]) were extracted by Biospin Virus RNA Extraction Kit (Hangzhou Bioer Technology Co., Ltd., China), and was electrophorethically fractionated for 6 h on a 10% polyacrylamide gel at 90 volts. The electrophoresis bands of nucleic acid were detected by Fast Sliver Stain Kit (Real Times Bio-technology Co., Ltd., China).

RVA replication was confirmed by indirect immunofluorescence assay (IFA) with a rabbit polyclonal antibody (orb241327, Biorbyt, Cambridge, UK) to RVA intermediate capsid protein VP6 and fluorescein-conjugated goat anti-rabbit IgG (H + L) (ZSGB-BIO, Beijing, China) as the primary and secondary antibodies, respectively. Growth curves of N4006 in adapted MA104 cells were generated by infecting fresh MA104 cell monolayers with RVA at a multiplicity of infection (MOI) of 0.01. Cell lysates after repeated freeze-thawing were collected every 12 h during 72 h of incubation, and the titers were determined by the 3,3’-diaminobenzidine (DAB) colorimetric method.

### Nuclear acid extraction and whole-genome sequencing

The whole-genome sequences of N4006 in the original fecal sample (P0) and the isolated strain at passage 10 (P10) were determined. According to the manufacturer’s instructions, viral RNA was extracted from the stool sample and MA104 cell culture using the Viral Nucleic Acid Extraction Kit (Xi’an Tianlong Technology Co., Ltd., China) and Nucleic Acid Automatic Extractor (NP968, Tianlong).

Using the One-Step RT-PCR Kit (Qiagen, Hilden, Germany), RT-PCR was conducted to amplify the 11 gene segments of N4006 using end-specific primers [20]. The reverse transcription was performed at 50 °C for 30 min, followed by PCR amplification at 95 °C for 15 min, 35 cycles, and a final extension of 8 min at 72 °C in a 5331 PCR Master Cycler (Eppendorf, Germany). The reaction conditions were 30 s at 94 °C, 30 s at 55 °C, and 3 min at 72 °C. Nucleic acid electrophoresis was performed in a 1.5% agarose gel, and the amplified product was detected using a Bio-Rad Universal Hood II Gel Doc System (Bio-Rad, Hercules, CA, USA). Strips of the amplification products were purified using an E.Z.N.A. Gel Extraction Kit (Omega Bio-tek, Norcross, GA, USA).

The purified amplification products were cloned in T plasmids to obtain accurate terminal sequences. Purified amplified products were ligated into pClone 007 vector at room temperature for 30 min (pClone 007 Vector Kit, Beijing TsingKe Biotech Co., Ltd., China) and transformed into chemically competent Trelief 5α cells (Beijing TsingKe Biotech Co., Ltd.). The transforming reagent was plated on LB plates containing ampicillin (Amp^r^) and incubated for 12–16 h in an incubator at 37℃. Selected monoclonal colonies were sent to Beijing TsingKe Biotech Co., Ltd. for direct sequencing.

### Phylogenetic and genetic analyses

Nucleotide sequence alignments and identifications were performed with DNASTAR software. The genotypes of all segments were determined using the RotaC tool, a web-based tool for the complete genome classification of RVA [21]. Maximum-likelihood phylogenetic trees for the 11 genes were constructed using MEGA ver. 5.0.5, with best fit models and 1000 bootstrap replicates. The following models were found to fit the indicated genes best: HKY + G + I (VP1), TN93 + G + I (VP2, VP4), T92 + G + I (VP3, NSP1), T92 + G (VP6, NSP4, NSP5), T92 + G + I (VP7), T92 + I (NSP2), and TN93 + I (NSP3). Reference sequences of recently circulating strains and vaccine strains were downloaded from GenBank. Lineage designations were defined based on previous studies [22–25].

### Deduced amino acid sequence alignments and structure-based predictions

Deduced amino acid sequence alignments and identity analysis were performed with BioEdit ver. 7.0.9.0. To assess the representativeness of N4006 as a vaccine strain and its differences from current vaccine strains, the presumed neutralizing epitopes on VP7 (7-1a, 7-1b, 7 − 2) and VP4 (VP8*: 8 − 1, 8 − 2, 8 − 3, 8 − 4; VP5*: 5 − 1, 5 − 2, 5 − 3, 5 − 4, 5–5) in N4006 were compared with those in Chinese or global epidemic strains and vaccines using BioEdit ver. 7.0.9.0. Structural analysis of VP7 and VP4 was conducted in PyMOL ver. 2.5.2 with the protein data bank files 4V7Q [26], 3FMG [27], and 1KQR [28].

## Results

### Isolation of RVA N4006

**Fig. 1:**
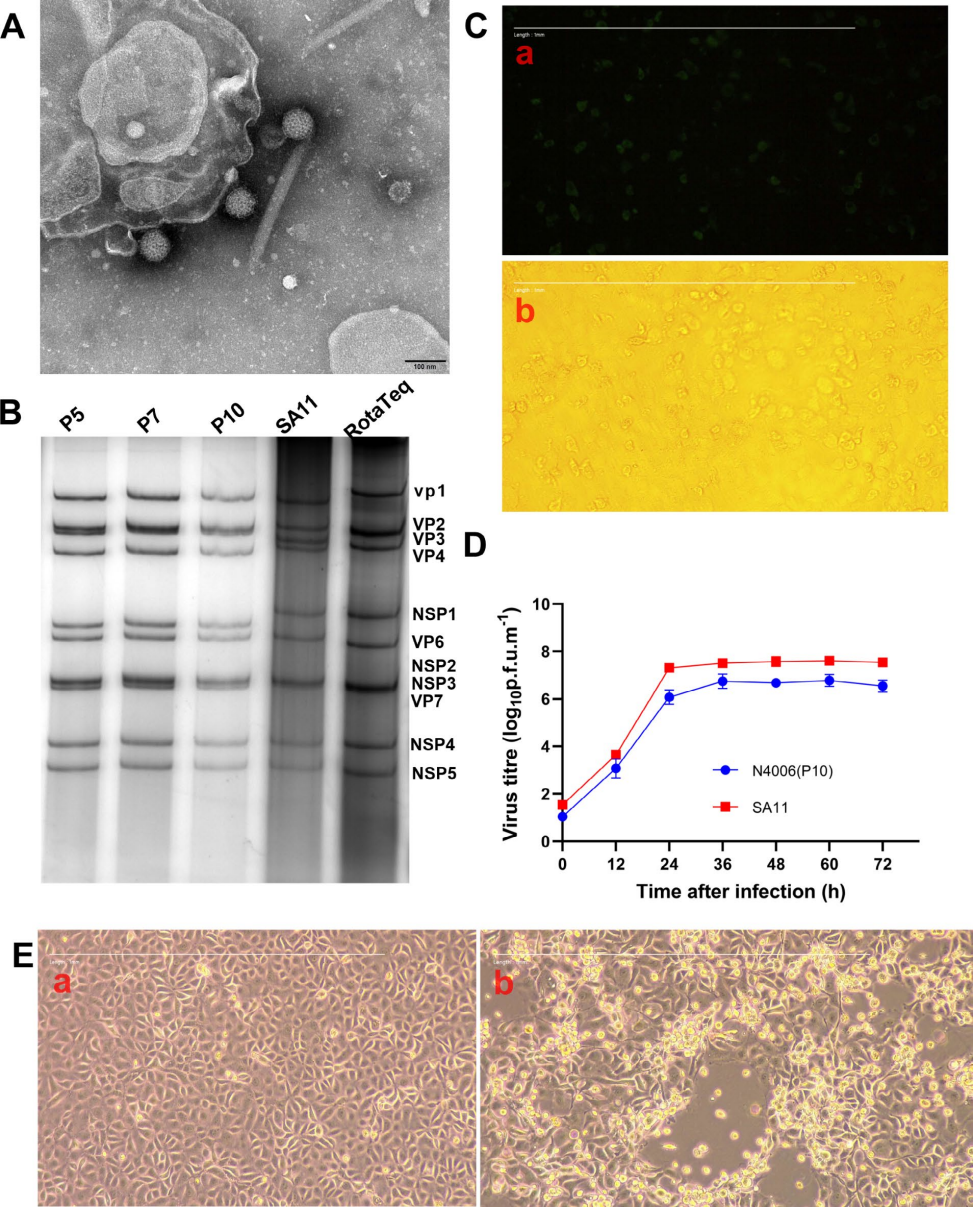
Characteristics of N4006 cultured in MA104 cells. (**A**) Morphology of RVA by TEM. (**B**) PAGE map of dsRNA genomic fragments of RVA strains SA11, RotaTeq W179-9 and N4006 of different generations. (**C**) Green fluorescence (20× magnification) was detected in MA104 cells at 24 h after infection with N4006. a, green fluorescence diagram of MA104 cells infected with N4006; b, MA104 cells infected with N4006 using light microscopy. (**D**) Growth curve of N4006. MA104 monolayer cells were infected with N4006 and SA11 (MOI 0.01). Cell lysates after repeated freeze-thawing were collected every 12 h, and titers were determined by DAB colorimetry. Results are the means of three determinations; error bars indicate standard deviation. (**E**) The CPE induced by RVA N4006 in MA104 cells. a, MA104 cells infected with N4006 with CPE; b, MA104 cells uninfected with rotavirus

After 10 passages with limiting dilution and plaque purification, RVA strain N4006 with G9P[8] genotype was isolated in MA104 cells. Based on TEM, the infected MA104 cells contained RVA particles with a 72–74 nm diameter, exhibiting a typical spoke form of rotavirus (Fig. 1A).

By PAGE and silver staining, the N4006 strain had a long RNA migration pattern 4:2:3:2 typical of human RVA (Fig. 1B). Additionally, the N4006 showed a unique RNA map, different from SA11 (G3P[3]) and RotaTeq W179-9 (G1P[5]). RVA replication was confirmed by IFA detection of VP6-specific epitopes (Fig. 1C). RVA N4006 (P10) titer peaked (10^5.5^ PFU/mL) at 48 h post-infection, and the simian rotavirus strain SA11 (10^7.5^ PFU/mL) at 24 h post-infection (Fig. 1D). It was not until the 6th passage that evident CPE, which included cell separation, cell fusion, cell shrinkage, cell rounding, cell accumulation, and cell lysis, was observed (Fig. 1E).

### Whole-genome sequencing

Alignment of full-length sequences showed that the sequences of N4006 from the original fecal sample and cell culture at passage 10 were identical. According to RotaC, the genomic constellation of N4006 was G9-P[8]-I1-R1-C1-M1-A1-N1-T1-E2-H1 (G9P[8]-E2). This atypical genotype that contained an NSP4 gene segment of the DS-1-like RVA G2P based on the Wa-like G9P genotype. All obtained genomic sequences were deposited in GenBank (accession numbers OP901804- OP9018014).

### Phylogenetic and genetic analyses

Nucleotide comparison between N4006 with RVA G9P[8]-E2 and G9P[8]-E1 from China and RVA G9P[8]-E2, G9P[8]-E1 and G2P[4] from Japan showed that the highest identities existed in RVA G9P[8]-E2 from both China and Japan for 10 of the genes (Table 1). NSP3 and NSP5 gene fragments showed 100% similarity in some RVA G9P[8]-E2 from China and Japan (Table 1). It is noteworthy that NSP4 showed 100% similarity in some RVA G9P[8]-E2 and G2P[4] from Japan and G9P[8]-E2 from China.

**Table 1:** Nucleotide sequence identities (%) when comparing N4006 with RVA G9P[8]-E2, G9P[8]-E1 and G2P[4] from China and Japan

strain	N1	N2	N3	N4	N5	V1	V2	V3	V4	V6	V7
CHN/E6398/2019/G9P8-E2	98.1	**99.9**	**100**	99.9	**100**	**99.9**	**99.9**	**99.8**	99.7	**97.4**	**99.9**
CHN/Fuzhou18-97/2018/G9P8-E2	98.1	99.9	**100**	99.9	99.7	99.8	99.7	99.6	99.7	97.4	99.5
CHN/Z2768/2019/G9P8-E2	**98.2**	99.8	99.9	**100**	**100**	99.9	99.9	99.8	**99.8**	97.4	99.9
CHN/L2448/2019/G9P8-E2	97.9	99.9	99.8	99.9	**100**	99.8	99.6	99.7	99.7	97.4	99.7
CHN/SZ18442015/2018/G9P8-E2	97.9	99.9	**100**	99.7	**100**	99.9	99.7	99.7	99.7	97.3	99.8
CHN/SZ18442205/2018/G9P8-E2	98.1	99.8	**100**	99.9	**100**	99.9	99.5	99.6	99.5	96.8	99.6
CHN/Fuzhou19-58/2019/G9P8-E1	97.8	**99.6**	99.7	83.6	99.3	**99.5**	**98.4**	**99.4**	**99.7**	**97.8**	**99.4**
CHN/Fuzhou19-84/2019/G9P8-E1	**98.1**	98.9	99.2	**83.8**	99.3	98.4	98.4	98.3	97.6	96.8	92.4
CHN/LL09131481-2009/G9P8/E1	97.2	99	**99.9**	82.9	97.5	10.6	21.7	22.7	36.1	99.2	93.2
JPN/Tokyo18-27/2018/G9P8-E2	96.8	99.8	99.9	99.9	**100**	99.8	**99.8**	99.6	99.6	**97.4**	99.7
JPN/Tokyo18-38/2018/G9P8-E2	**98.2**	99.7	99.9	**100**	**100**	**99.9**	99.7	**99.7**	**99.7**	97.3	99.7
JPN/Tokyo18-30/2018/G9P8-E2	97.9	99.7	99.7	99.7	**100**	99.8	99.7	99.7	99.7	97.4	**99.8**
JPN/Tokyo18-43/2018/G9P8-E2	97.7	**99.9**	**100**	99.9	**100**	99.9	99.7	99.7	99.7	97.4	99.8
JPN/Tokyo18-50/2018/G9P8-E1	97.1	99.8	99.7	**83.9**	**100**	**99.8**	**99.8**	98.2	99.7	97.4	**99.7**
JPN/Tokyo18-37/2018/G9P8-E1	97.9	98.9	**99.8**	83.9	99	98	98.1	98.2	99.3	**98.7**	92.8
JPN/Tokyo16-4754/2017/G9P8-E1	97.9	**99.9**	99.1	83.6	99.3	99.6	99.7	**99.4**	**99.6**	97.7	99.5
JPN/Tokyo16-3571/2017/G9P8-E1	98.1	99.8	97.7	83.6	99.3	99.7	99.7	99.4	99.5	97.7	99.6
JPN/Tokyo17-15/2017/G2P4	74.4	83.3	78.3	99.9	83.8	79.6	79.3	77.6	86.4	79.6	74.6
JPN/Tokyo17-17/2017/G2P4	75.6	83	78.3	99.6	83.7	79.8	79.7	77.1	86.3	79.5	74.5
JPN/YR116/2013/G2P4	75.8	83.2	78.4	**100**	81.3	79.8	79.6	77.1	86.6	79.4	74.6
JPN/KN161/2014/G2P4	75.6	83.1	78.4	**100**	83.7	79.7	79.6	77.2	86.6	79.4	74.4

Phylogenetic trees were constructed for the 11 gene fragments of N4006 (P0) (Fig. 2). VP7 genes of G9 RVAs were divided into six lineages in the phylogenetic tree of VP7 (Fig. 2A). The global G9-VP7 genes were mainly distributed in lineages III and VI, whereas other lineages encompassed older strains and vaccines. Although Chinese G9 strains were mainly in the III and VI lineages, N4006 and most G9 RVAs circulating in China in recent years were in G9-lineage VI. N4006 was closest to the G9P[8]-E2 epidemic strains of 2019 and 2018 in China and Japan, such as Z2768, E6398, JZ1810, and Tokyo18-43. By contrast, N4006 was far from the G9 vaccine strain ROTAVAC-116E (lineage II) and the G9 vaccine strain ROTASIIL-Au32 (G9-lineage I), with 88.5% and 89.0% nucleotide sequence identities, and 92.4% and 94.5% aa identities, respectively. The VP7 identity between N4006 and sequences of lineage VI were 91.7–99.9% nt and 98.5–100% aa. The identities of N4006 and sequences of lineage III were 85.3–99.5% nt, and 92.2–99.7% aa. The VP7 identities between N4006 and other lineages were 82.3–90.6% nt, and 92.4–96.3% aa.

**Fig. 2:**
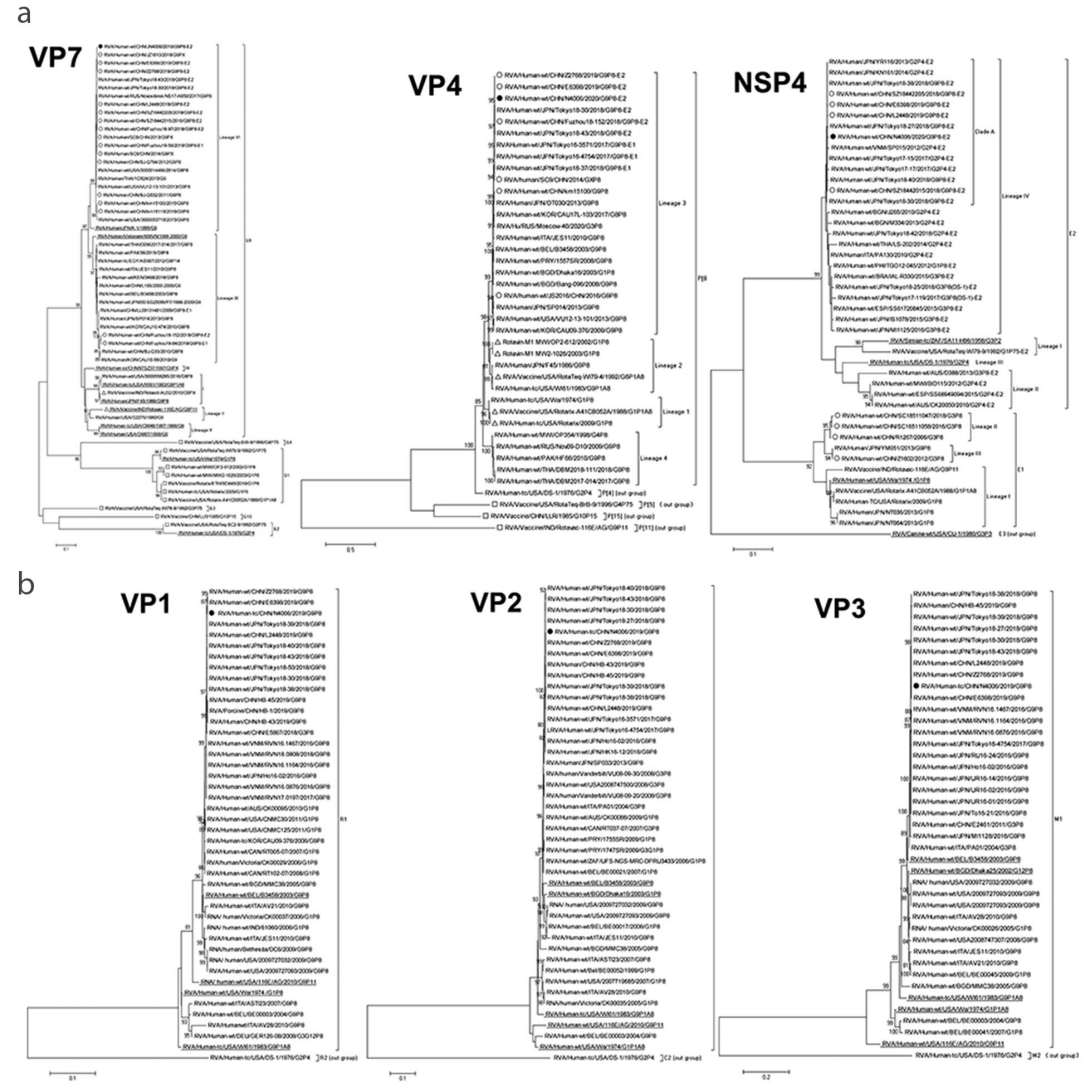
Maximum-likelihood phylogenetic trees of G9P[8] RVA N4006. Bootstrap values (1000 replicates) are shown at branch nodes, and values < 80% are hidden. Scale bars indicate distance. Black solid circular pattern, G9P[8] strain N4006. Underlined sequences, prototype strains. (**A**) VP7 phylogeny of G9, VP4 phylogeny of P[8] and NSP4 phylogeny. “O” Chinese human RVA sequences from the indicated years. “Δ” vaccines with the same genotype as N4006. “□” vaccines of other genotypes. (**B**) VP1-VP3 phylogeny. (**C**) VP6, NSP1 and NSP2 phylogeny. (**D**) NSP3 and NSP5 phylogeny

**Fig. 2:**
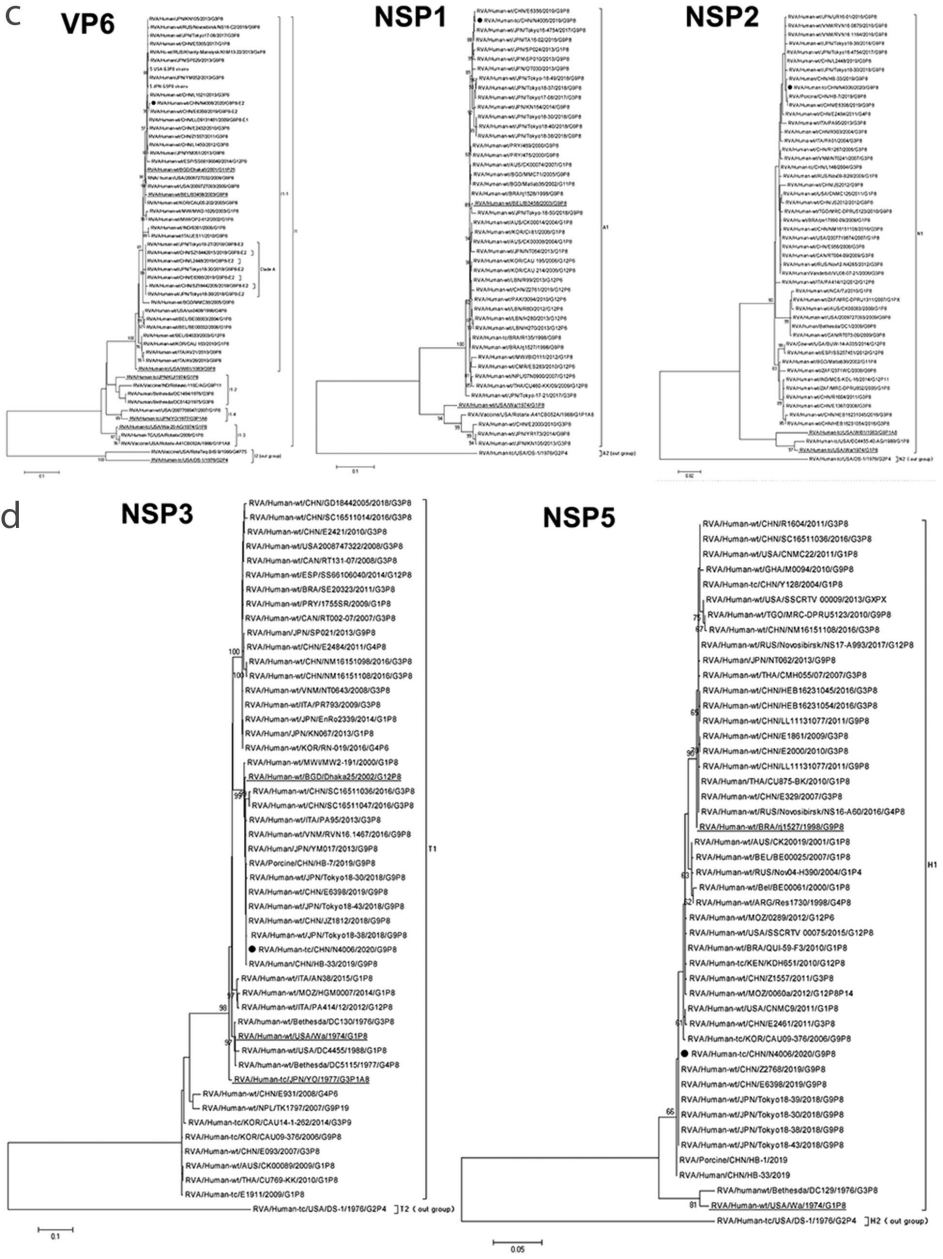
(continued)

The VP4 genes of P[8] RVAs were divided into four lineages [29] (Fig. 2A). Most contemporary P[8] RVAs, including G3P[8], G9P[8], and G1P[8] strains from all over the world were clustered into P[8]-lineage 3, sharing 94.6–99.8% nt and 97.0–99.6% aa sequence identities with that of N4006. The VP4 gene of N4006 was closest to that of 2019 Chinese G9P[8] viruses (e.g., Z2768 and Z6398), showing 99.6–99.8% nt and 99.4–99.6% aa sequence identities, and was close to that of 2018 Japanese G9P[8]-E2 strains (e.g., Tokyo18-30, Tokyo18-152, and Tokyo18-43), with 98.6–99.7% nt and 99.1–99.8% aa sequence identities. The VP4 sequence similarities between N4006 and the P[8] genotype vaccines RotaTeq (strain RotaTeq-W179-4), Rotarix (strains Rotarix and Rotarix-A41CB052A ), and Rotavin-M1(strains OP2-612 and MW2-1026) were 88.2–92.5% nt and 93.3–95.5% aa. In the VP4 phylogenetic tree, N4006 was also dissimilar to the latter strains.

In the phylogenetic tree of NSP4 fragments of N4006 and other reference strains (Fig. 2A), NSP4 belonged to the E1 and E2 genotypes. Chinese E1 RVAs were of G3P[8] and G9P[8] genotypes. There were four lineages in genotype E2. The prevalent viruses including N4006 were mainly distributed in lineage E2-IV, closest to Chinese and Japanese RVA G9P[8]-E2 emerging in 2018 or 2019. They were clustered into a small sub-branch named clade A together with DS-1-like G2P[4] strains from Japan and Vietnam (e.g., Tokyo17-17, SP015, and Tokyo17-15). The identities of sequences of clade A were 94.5–100% nt and 97.7–100% aa. There are few G2P[4] RVAs in China. G1P[8], G3P[8], and G2P[4] genotype RVAs from Japan, Bulgaria, Italy, the Philippines, Brazil, Spain, and Thailand comprised the rest of lineage E2-IV and included reassortant G3P[8]-E2 RVA strains (e.g., Tokyo18-25, IS1078, Tokyo17-119, and MI1125) in Japan from 2015 to 2018.

Phylogenetic trees were constructed for the other eight gene fragments of N4006 (Fig. 2B–D). N4006 was closest to 2019 Chinese G9P[8]-E2 viruses (e.g., Z2768, E6398 ,L2448, E6356, HB-45, and HB-43), and close to 2018 Japanese G9P[8]-E2 strains (e.g., Tokyo 18–39, Tokyo 18–40, Tokyo 18–43, Tokyo 18–30, and Tokyo 18–38). The identities among these E2 strains were in the range of 94.6–100% nt and 98.0–100% aa.

### Sequence alignments and structure-based predictions

We compared the presumed neutralizing epitopes on VP7 and VP4 (VP5* and VP8*) of N4006 with those of other G9 epidemic strains and vaccines (Tables 2 and 3).

### Presumed VP7 antigenic epitope

The VP7 neutralizing antigen epitope analysis (Table 2) suggested no more than one aa variation between N4006 and G9 epidemic strains in lineage VI of the VP7 phylogenetic tree, and no more than two aa variations between N4006 and G9 epidemic viruses in lineage III. N4006 belonged to lineage VI, which encompassed most Chinese epidemic RVA.

**Fig. 3:**
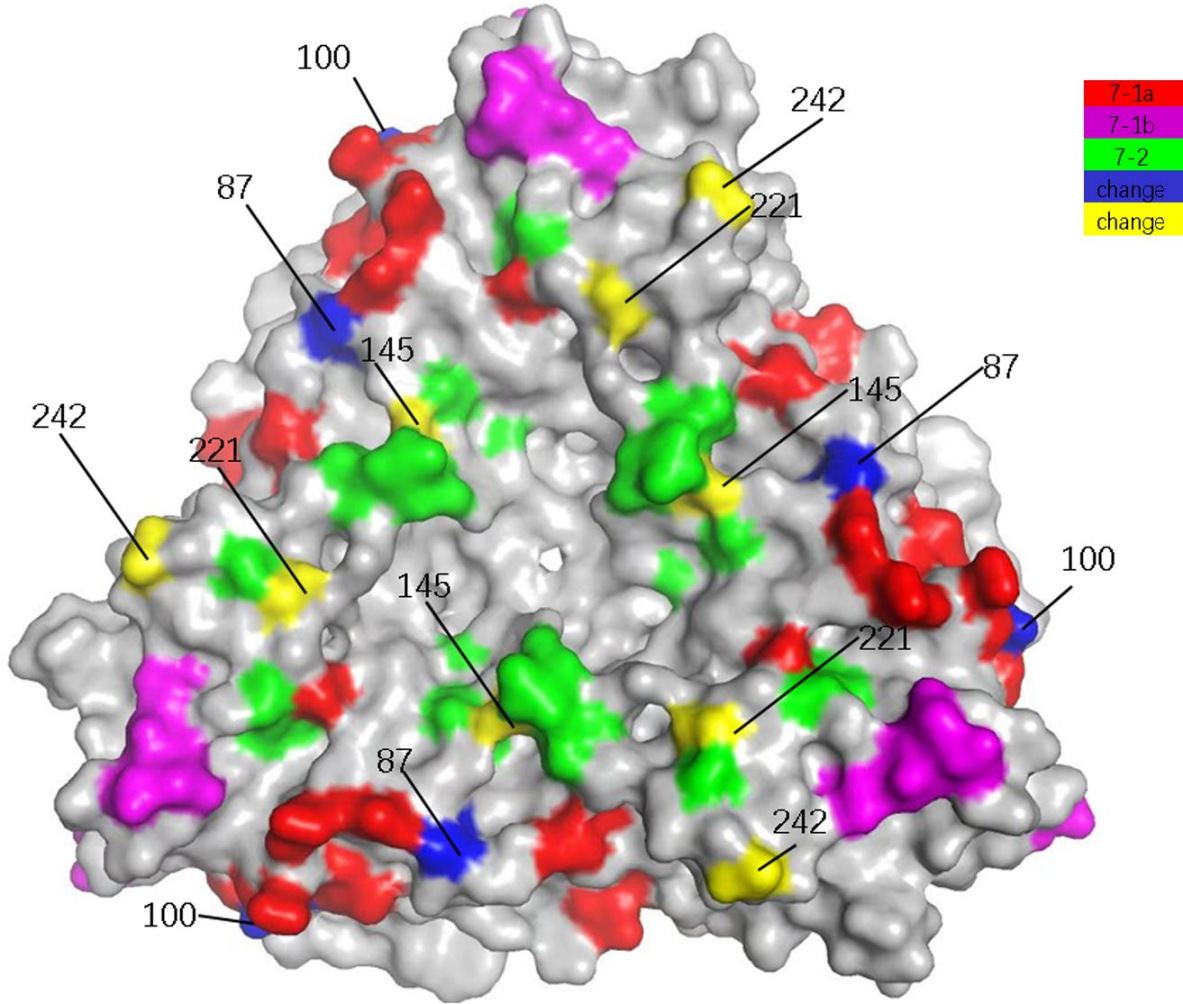
Presumed VP7 surface exposure antigen epitope amino acid variations between N4006 and ROTASIIL and ROTAVAC vaccine strains. VP7 trimer with surface-exposed antigen epitopes 7-1a (red), 7-1b (magenta), and 7 − 2 (green) (PDB 3FMG) are shown [27]. VP7 surface-exposed antigen epitopes amino acid variations between N4006 and the ROTASIIL and ROTAVAC vaccine strains on VP7 protein shown. Amino acid residues in N4006 that differed from both vaccine strains are in blue, and residues that differed from one of the vaccines are in yellow

There were three aa variations between the G9-genotype vaccine ROTASIIL (Serum Institute, India) and N4006 (Table 2; Fig. 3). These sites were in 7-1a and 7-1b. Of them, A87T and D100N might affect the immunogenicity of the vaccine. Compared with ROTASIIL, all G9P[8] epidemic strains had T242N (Fig. 3). All pedigree VI-G9P[8] epidemic strains had A87T and D100N. Most lineage III-G9P[8] epidemic strains had the same 100D as ROTASIIL, and a few had D100N (Table 2). The vast majority of lineage III-G9P[8] epidemic strains had 87T, unlike ROTASIIL, and a few had 87 A (like ROTASIIL) (Table 2).

Four aa variations existed between the G9-genotype vaccine ROTAVAC (Bharat Biotech, India) and N4006 (Table 2; Fig. 3). These sites were in 7-1a and 7 − 2, among which A87T, D100N, and N145D exhibited features of escape mutants. Compared with ROTAVAC, all G9P[8] circulating strains had N145D (Fig. 3). All G9P[8] epidemic strains in lineage VI had A87T, D100N, and N221S. Most G9P[8] epidemic strains in lineage III had 87T and 100D (unlike ROTAVAC), and a few had 87 A and 100 N. More than half in lineage III had 221 S, and a few had 221G, unlike ROTAVAC (Fig. 3). T96A and A125T were present in some strains in lineage VI (Table 2). Comparison with the VP7 putative epitopes of existing vaccines with other G genotypes (RV1 (G1), ROTAVIN-M1 (G1), RV5 (G1, G2, G3, G4), and LLR(G10)) indicated 12–16 aa variations in N4006 (Table 2).

**Table 2:** Presumed VP7 neutralizing antigen epitope differences between N4006 and strains used in vaccines or G9 strains circulating globally

Strains	7-1a														7-1b						7 − 2								
	**87**	**91**	**94**	**96**	**97**	**98**	**99**	**100**	**104**	123	125	129	130	291	**201**	**211**	212	**213**	**238**	242	143	**145**	146	**147**	**148**	**190**	**217**	221	**264**
N4006	T	T	G	T	E	W	K	N	Q	D	A	I	D	K	Q	N	T	A	D	N	K	D	S	T	L	S	E	S	G
E6398/2019/CHN/G9-E2 ^*a*^	T	T	G	T	E	W	K	N	Q	D	A	I	D	K	Q	N	T	A	D	N	K	D	S	T	L	S	E	S	G
Fuzhou19-58/2019/CHN/G9-E1 ^*a*^	T	T	G	T	E	W	K	N	Q	D	A	I	D	K	Q	N	T	A	D	N	K	D	S	T	L	S	E	S	G
JZ1810/2018/CHN/G9 ^*a*^	T	T	G	T	E	W	K	N	Q	D	A	I	D	K	Q	N	T	A	D	N	K	D	S	T	L	S	E	S	G
Km15119/2016/CHN/G9 ^*a*^	T	T	G	T	E	W	K	N	Q	D	**T**	I	D	K	Q	N	T	A	D	N	K	D	S	T	L	S	E	S	G
Km15100/2015/CHN/G9 ^*a*^	T	T	G	T	E	W	K	N	Q	D	**T**	I	D	K	Q	N	T	A	D	N	K	D	S	T	L	S	E	S	G
SC8/2014/CHN/G9 ^*a*^	T	T	G	**A**	E	W	K	N	Q	D	A	I	D	K	Q	N	T	A	D	N	K	D	S	T	L	S	E	S	G
SC6/2013/CHN/G9 ^*a*^	T	T	G	T	E	W	K	N	Q	D	A	I	D	K	Q	N	T	A	D	N	K	D	S	T	L	S	E	S	G
BJ-Q794/2012/CHN/G9 ^*a*^	T	T	G	T	E	W	K	N	Q	D	A	I	D	K	Q	N	T	A	D	N	K	D	S	T	L	S	E	S	G
BJ-Q532/2011/CHN/G9 ^*a*^	T	T	G	T	E	W	K	N	Q	D	A	I	D	K	Q	N	T	A	D	N	K	D	S	T	L	S	E	S	G
Tokyo18-43/2018/JPN/G9-E2 ^*a*^	T	T	G	T	E	W	K	N	Q	D	A	I	D	K	Q	N	T	A	D	N	K	D	S	T	L	S	E	S	G
NS17-A959/2017/RUS/G9 ^*a*^	T	T	G	T	E	W	K	N	Q	D	A	I	D	K	Q	N	T	A	D	N	K	D	S	T	L	S	E	S	G
3,000,053,718/2015/USA/G9 ^*a*^	T	T	G	T	E	W	K	N	Q	D	**T**	I	D	K	Q	N	T	A	D	N	K	D	S	T	L	S	E	S	G
3,000,014,466/2014/USA/G9 ^*a*^	T	T	G	T	E	W	K	N	Q	D	A	I	D	K	Q	N	T	A	D	N	K	D	S	T	L	S	E	S	G
1CR24/2015/THA/G9 ^*a*^	T	T	G	T	E	W	K	N	Q	D	A	I	D	K	Q	N	T	A	D	N	K	D	S	T	L	S	E	S	G
Fuzhou19-84/2019/CHN/G9-E1 ^*b*^	T	T	G	T	E	W	K	**D**	Q	D	A	I	D	K	Q	N	T	A	D	N	K	D	S	T	L	S	E	**G**	G
Fuzhou18-152/2018/CHN/G9-E2 ^*b*^	T	T	G	T	E	W	K	**D**	Q	D	A	I	D	K	Q	N	T	A	D	N	K	D	S	T	L	S	E	**G**	G
BJ-Q33/2010/CHN//G9 ^*b*^	T	T	G	T	E	W	K	**D**	Q	D	A	I	D	K	Q	N	T	A	D	N	K	D	S	T	L	S	E	**G**	G
LL09131481/2009/CHN/G9P8-E1 ^*b*^	T	T	G	T	E	W	K	**D**	Q	D	A	I	D	K	Q	N	T	A	D	N	K	D	S	T	L	S	E	S	G
L169/2000–2006/CHN/G9 ^*b*^	T	T	G	T	E	W	K	**D**	Q	D	A	I	D	K	Q	N	T	A	D	N	K	D	S	T	L	S	E	S	G
CAU10-55/2010/KOR/G9 ^*b*^	T	T	G	T	E	W	K	**D**	Q	D	A	I	D	K	Q	N	T	A	D	N	K	D	S	T	L	S	E	**G**	G
SP014/2013/JPN/G9 ^*b*^	T	T	G	T	E	W	K	**D**	Q	D	A	I	D	K	Q	N	T	A	D	N	K	D	S	T	L	S	E	S	G
3468/2016/KEN/G9 ^*b*^	T	T	G	T	E	W	K	**D**	Q	D	A	I	D	K	Q	N	T	A	D	N	K	D	S	T	L	S	E	S	G
3,000,558,285/2016/USA/G9P8 ^*b*^	**A**	T	G	T	E	W	K	**D**	Q	D	A	I	D	K	Q	N	T	A	D	N	K	D	S	T	L	S	E	S	G
ITA/JES11/2010/G9P8 ^*b*^	T	T	G	T	E	W	K	N	Q	D	A	I	D	K	Q	N	T	A	D	N	K	D	S	T	L	S	E	S	G
Rotasiil-AU32/G9 ^*c*^	**A**	T	G	T	E	W	K	**D**	Q	D	A	I	D	K	Q	N	T	A	D	**T**	K	D	S	T	L	S	E	S	G
Rotavac-116E/G9 ^*c*^	**I**	T	G	T	E	W	K	**G**	Q	D	A	I	D	K	Q	N	T	A	D	N	K	**N**	S	T	L	S	E	**N**	G
RV1/G1 ^*d*^	T	T	**N**	**G**	E	W	K	**D**	Q	S	**V**	**V**	D	K	Q	N	**V**	**D**	**N**	**T**	K	D	**Q**	**N**	L	S	**M**	**N**	G
Rotavin-M1/G1 ^*d*^	T	T	**S**	**G**	E	W	K	**D**	Q	**N**	**V**	**V**	D	**R**	Q	N	**V**	**D**	**N**	**T**	K	D	**Q**	**N**	L	S	**T**	**N**	G
RV5/G1 ^*d*^	T	T	**N**	**G**	**D**	W	K	**D**	Q	S	**V**	**V**	D	K	Q	N	**V**	**D**	**N**	**T**	K	D	**Q**	**S**	L	S	**M**	**N**	G
RV5/G2 ^*d*^	**A**	**N**	**S**	**D**	E	W	E	N	Q	D	**T**	**M**	**N**	K	Q	**D**	**V**	**S**	**N**	**S**	R	D	**N**	T	**S**	**D**	**I**	S	G
RV5/G3 ^*d*^	T	T	**N**	**N**	**S**	W	K	**D**	Q	D	A	**V**	D	K	Q	**D**	**A**	**N**	**K**	**D**	K	D	**A**	T	L	S	E	**A**	G
RV5/G4 ^*d*^	**S**	T	**S**	T	E	W	K	**D**	Q	**N**	**L**	I	D	K	Q	**D**	T	A	D	**T**	**R**	**A**	S	**G**	**E**	S	**T**	S	G
LLR/G10 ^*d*^	T	T	**N**	**N**	E	W	**T**	**S**	Q	**N**	A	**V**	D	K	Q	N	T	**G**	D	**T**	**R**	**N**	S	**S**	L	S	E	**A**	G

VP7 presumed neutralizing antigen epitopes (7-1a, 7-1b, 7 − 2) of N4006 were compared with vaccine strains and worldwide RVA G9 epidemic strains. ^*a*^ Epidemic strains of lineage VI in the VP7 phylogenetic tree; ^*b*^ epidemic strains of lineage III; ^*c*^ G9 genotype vaccines; ^*d*^ vaccines of other G genotypes; bold numbers, antigen-escape-related neutralizing epitopes; bold letters, Changes in antigen epitopes of reference strains compared with N4006

### Presumed VP4 antigenic epitopes

**Table 3:** Presumed VP4 neutralizing antigen epitope differences between N4006 and vaccines or P[8] strains circulating globally

Strains	8 − 1											8 − 2		8 − 3									8 − 4			5 − 1								5 − 2	5 − 3	5 − 4	5–5
	**100**	**146**	**148**	**150**	**188**	**190**	192	193	**194**	195	196	**180**	**183**	113	**114**	115	**116**	125	131	**132**	**133**	**135**	**87**	**88**	**89**	**384**	**386**	**388**	**393**	**394**	**398**	**440**	**441**	**434**	**459**	**429**	**306**
N4006	D	S	Q	D	S	T	N	L	N	G	I	T	A	D	P	V	D	N	R	N	D	D	N	T	N	Y	F	I	W	P	G	R	T	P	E	L	R
Z6398/2019/CHN/G9P[8]-E2 ^*a*^	D	S	Q	D	S	T	N	L	N	G	I	T	A	D	P	V	D	N	R	N	D	D	N	T	N	Y	F	I	W	P	G	R	T	P	E	L	R
Fuzhou18-152/2018/CHN/G9P[8] ^*a*^	D	S	Q	D	S	T	N	L	N	G	I	T	A	D	P	V	D	N	R	N	D	D	N	T	N	Y	F	I	W	P	G	R	T	P	E	L	R
JS2016/2016/CHN/G9P[8] ^*a*^	D	S	Q	D	S	T	N	L	N	G	I	T	A	D	P	V	D	N	R	N	D	D	N	T	N	Y	F	I	W	P	G	R	T	P	E	L	R
Km15100/2015/CHN/G9P[8] ^*a*^	D	S	Q	D	S	T	N	L	N	G	I	T	A	D	P	V	D	N	R	N	D	D	N	T	N	Y	F	I	W	P	G	R	T	P	E	L	R
SC9/2014/CHN/G9P[8] ^*a*^	D	S	Q	D	S	T	N	L	N	G	I	T	A	D	P	V	D	N	R	N	D	D	N	T	N	Y	F	I	W	P	G	R	T	P	E	L	R
Tokyo18-43/2018/JPN/G9P[8] ^*a*^	D	S	Q	D	S	T	N	L	N	G	I	T	A	D	P	V	D	N	R	N	D	D	N	T	N	Y	F	I	W	P	G	R	T	P	E	L	R
VU12-13-101/2013/USA/G9P[8] ^*a*^	D	S	Q	D	S	T	N	L	N	G	I	T	A	D	P	V	D	N	R	N	D	D	N	T	N	Y	F	I	W	P	G	R	T	P	E	L	R
JES11/2010/ITA/G9P[8] ^*a*^	D	S	Q	D	S	T	N	L	N	G	I	T	A	**N**	P	V	D	N	R	N	D	D	N	T	N	Y	F	I	W	P	G	R	T	P	E	L	R
CAU09-376/2017/KOR/G9P[8] ^*a*^	D	S	Q	D	S	T	N	L	N	**D**	I	T	A	D	P	V	D	N	R	N	D	D	N	T	N	Y	F	I	W	P	G	R	T	P	E	L	R
Moscow-40/2020/RUS/G3P[8] ^*b*^	D	S	Q	D	S	T	N	L	N	G	I	T	A	D	P	V	D	N	R	N	D	D	N	T	N	Y	F	I	W	P	G	R	T	P	E	L	R
Dhaka16/BGW/2003/G1P[8] ^*b*^	D	S	Q	D	S	T	N	L	N	G	I	T	A	D	P	V	D	N	R	N	D	D	N	T	N	Y	F	I	W	P	G	R	T	P	E	L	R
CAU17L-103/2009/KOR/G8P[8] ^*b*^	D	S	Q	D	S	T	N	L	N	G	I	T	A	D	P	V	D	N	R	N	D	**N**	N	T	N	Y	F	I	W	P	G	R	T	P	E	L	R
RV1/G1P[8] ^*c*^	D	S	Q	**E**	S	T	N	L	N	**N**	I	T	A	**N**	P	V	D	**S**	**S**	N	D	**N**	N	T	N	Y	F	I	W	P	G	R	T	P	E	L	R
RV5/P[8] ^*c*^	D	S	Q	**E**	S	T	N	L	N	**D**	I	T	A	**N**	P	V	D	N	R	N	D	D	N	T	N	Y	F	**L**	W	P	G	R	T	P	E	L	R
Rotavin-M1/P[8] ^*c*^	D	S	Q	**E**	S	T	N	L	N	**D**	I	T	A	**S**	P	V	D	N	R	N	D	D	N	T	N	Y	F	I	W	P	G	R	T	P	E	L	R
LLR/P[15] ^*d*^	D	**T**	**N**	**T**	**Y**	T	N	**Y**	**D**	**S**	**V**	T	A	**P**	**E**	**T**	**T**	**T**	**A**	N	**P**	**Q**	**T**	**S**	**E**	Y	F	**L**	W	P	G	R	T	P	**D**	L	R
Rotavac-116E/P[11] ^*d*^	**T**	S	**A**	**A**	**G**	**Y**	N	**V**	**P**	**N**	**A**	**D**	**G**	**A**	**Q**	**T**	**S**	**T**	**D**	N	**S**	**S**	**S**	**N**	**D**	Y	F	**L**	W	P	G	**G**	T	P	**Q**	**C**	R
RV5/P[5] ^*d*^	**G**	**A**	**D**	D	**Y**	**V**	N	**Y**	**A**	**S**	**V**	T	A	**T**	**S**	**E**	**T**	**S**	**S**	N	**A**	D	**T**	**G**	**P**	Y	F	**L**	W	P	G	R	T	P	E	L	R

VP4 consists of two subunits, VP8* and VP5*, which have neutralizing epitope regions. The presumed neutralizing antigen epitopes in VP8* (8 − 1, 8 − 2, 8 − 3, 8 − 4) and VP5* (5 − 1, 5 − 2, 5 − 3, 5 − 4, 5–5) of N4006 were compared with those of vaccine strains and RVA P[8] epidemic strains. ^*a*^ Prevalent strains of G9P[8] genotype in VP4 phylogenetic tree lineage 3; ^*b*^ prevalent strains of P[8] of other G genotypes in lineage 3; ^*c*^P[8] genotype vaccines; ^*d*^ vaccines of other P genotypes; bold numbers, antigen-escape-related neutralizing epitopes; bold letters, changes in antigen epitopes of reference strains compared with N4006. For prevalent strains of P[8] belonging to other G genotypes in lineage 3, only a few strains were used as references

We also analyzed presumed antigen epitopes on VP4 (VP8* and VP5*) (Table 3). N4006 was almost identical not only to G9 but also to other G genotype Wa-like epidemic strains of P[8] genotype in China and worldwide, such as Moscow-40 (G3P[8]), Dhaka16 (G1P[8]), and CAU17L-103 (G8P[8]). All P[8] genotype Wa-like epidemic strains had almost identical aa sequences in the eight epitope regions (8 − 1, 8 − 2, 8 − 3, 8 − 4, 5 − 1, 5 − 2, 5 − 3, 5 − 4, and 5–5), except for a few with 195D, 113 N, and 135 N, which were different from the majority (195G, 113D, and 135D).

**Fig. 4:**
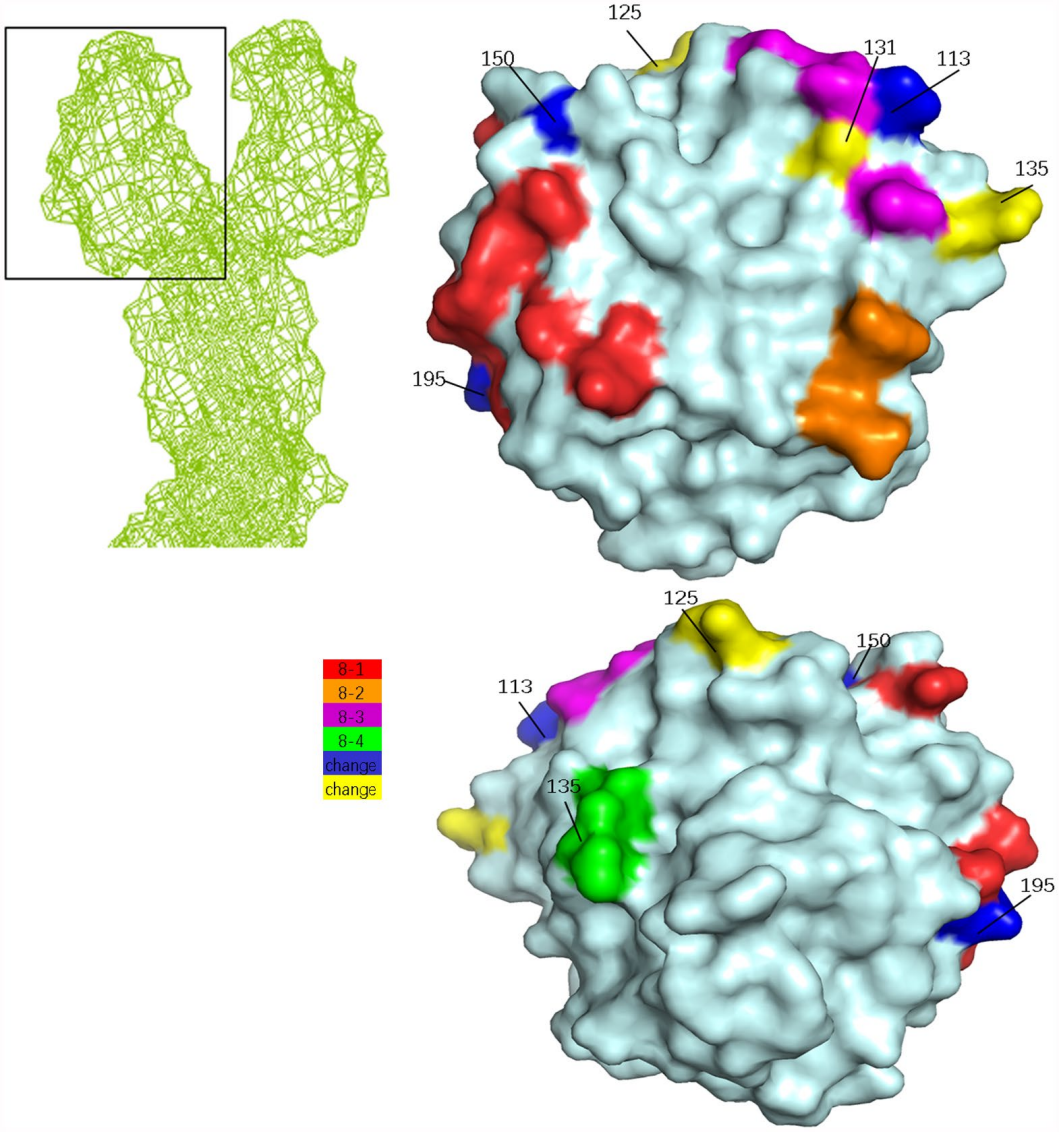
VP8^*^ presumed surface exposure antigen epitope amino acid residue variations between N4006 and the RV1, RV5, and ROTAVIN-M1 vaccine viruses. Top left corner, mesh diagram of RVA VP4; the VP8^*^ domain (PDB 4V7Q) is boxed [27]. The front (upper right corner) and back (lower right corner) of the enlarged VP8^*^ domain are shown, with the surface-exposed antigen epitopes 8 − 1 (red), 8 − 2 (orange), 8 − 3 (magenta), and 8 − 4 (green) (PDB 1KQR) [29]. VP8^*^ surface-exposed antigen epitope amino acid variations between N4006 and the RV1, RV5, and ROTAVIN-M1 vaccine viruses are shown. Amino acid residues in N4006 that differed from both vaccines are in blue, and residues that differed from one of the vaccines are in yellow

Compared with P[8] epidemic strains, there were six aa variations in P[8]-genotype vaccine RV1 in VP4 antigenic epitopes at sites 150, 195, 113, 125, 131, and 135 in epitope regions 8 − 1 and 8 − 3 (Table 3; Fig. 4). Sites 150, 195, 125, and 131 differed from the epidemic strains. Among the six differences, D150E and D135N in epitopes 8 − 1 and 8 − 3, might affect vaccine immunogenicity (Table 3).

Compared with P[8] epidemic strains, there were four aa variations in P[8]-RV5 at sites 150, 195, 113, and 388 in VP4 antigenic epitopes (Table 3; Fig. 4). Sites 150 and 388 were different from epidemic strains. D150E and I388L showed characteristics of escape mutants (Table 3).

There were three aa variations in the P[8]-genotype vaccine ROTAVIN-M1 at aa sites 150, 195, and 113 in VP4 antigenic epitopes (Table 3; Fig. 4). Sites 150 and 113 were different from the epidemic strains. D150E might show characteristics of escape mutants (Table 3).

Comparison with the VP4 putative epitopes of existing vaccines of other P genotypes such as LLR (P[15]), ROTAVAC (P[11]), and RV5 (P[5]) indicated 19–22 aa variations in N4006 (Table 3).

## Discussion

Rotavirus was first isolated by Wyatt and named Wa [30]. Laboratory rotavirus strains, such as SA11 and RRV, can be continuously propagated in cell lines to 10^7^–10^8^ PFU/mL. However, the success rate of RVA isolation from human diarrhea stool is low, and the titer of adaptive culture is lower than that of model rotaviruses [19]. In this study, RVA N4006 was isolated from a child aged 1 year, 3 months, and 24 days with diarrhea in MA104 cells at a high titer (10^5.5^ PFU/mL). RVA particles with a diameter of 70–80 nm are mature and can continue to infect new host cells. The 11 RNA fragments of RVA were separated by PAGE, and the aggregation mode of 4:2:3:2 was displayed. Rotaviruses with other aggregation patterns might be from birds or non-group A rotavirus, or have rearranged genes. N4006 had a nucleotide electrophoresis migration pattern identical to RVA, and the nucleotide electrophoresis migration patterns of different generations were identical, indicating the stability of its genetic genes. CPE in MA104 cells and in other rotavirus-sensitive cell lines like CV-1 and Caco-2, according to our previous study (Figure S1), showed that N4006 was isolated and propagated in these cells. The rotavirus titer was low after more than 10 generations of adaptation in MA104 cells [31]. The high titer of N4006 (10^5.5^ PFU/mL) with no adaptation mutation implies its adaptation to a high titer in subsequent passages. In addition, although the rotavirus was successfully isolated, the trypsin concentration of the culture medium used in this study is fixed doses (20ug/ml), which may not be the best, we will explore the best concentration in future studies.

The G9P[8] genotype is the fifth most common RVA epidemic genotype combination worldwide after G1P[8], G2P[4], G3P[8], and G4P[8] [32]. G9P[8] RVA as a cause of acute gastroenteritis in children was first reported in the United States in 1983 [33]. Although G9P[8] was later sporadically detected in Japan, Thailand, and other countries [34, 35], it was not until one or two decades later that G9P[8] RVA emerged in large numbers and spread worldwide [36–38]. In China, the first human RVA G9P[8] was isolated from a child with gastroenteritis in 1997 and named T203 [39]. Thereafter, the number of G9P[8] RVA strains increased rapidly [12–14], and was detected in 74.05% of hospitalized children with acute gastroenteritis in 2016 (data not shown). However, G9P[8] RVA strains before 2016 typically had the Wa-like gene constellation: I1-R1-C1-M1-A1-N1-T1-E1-H1(Table 4). Whole-genome sequence analysis showed that N4006 belonged to a recently emerged G9P[8]-E2 (G9-P[8]-I1-R1-C1-M1-A1-N1-T1-E2-H1). Because the E2 genotype NSP4 is present in DS-1-like RVA (Table 4), N4006 was considered a reassortment strain. The newly emerged G9P[8]-E2 was first reported in Japan in 2019 and became the prevalent strain locally [24] and spread widely in Tokyo. According to the China CDC Rotavirus monitoring network, G9P[8]-E2 has been the main RVA genotype combination in China since 2018 (data not shown). Therefore, N4006, which matches the most widespread genotype, represents RVAs in China.

**Table 4:** Whole genome constellation of N4006 and other human RVA

Strain	VP7	VP4	VP6	VP1	VP2	VP3	NSP1	NSP2	NSP3	NSP4	NSP5
N4006	G9	P[8]	I1	R1	C1	M1	A1	N1	T1	**E2**	H1
Wa-like RVA	G1	P[8]	I1	R1	C1	M1	A1	N1	T1	E1	H1
	G3	P[8]	I1	R1	C1	M1	A1	N1	T1	E1	H1
	G4	P[8]	I1	R1	C1	M1	A1	N1	T1	E1	H1
	G9	P[8]	I1	R1	C1	M1	A1	N1	T1	E1	H1
DS-1-like RVA	G2	P[4]	I2	R2	C2	M2	A2	N2	T2	E2	H2
Au-1-like RVA	G3	P[9]	I3	R3	C3	M3	A3	N3	T3	E3	H3
RVA G9P[8] before 2018 in China	G9	P[8]	I1	R1	C1	M1	A1	N1	T1	**E1**	H1
RVA G9P[8] after 2018 in China	G9	P[8]	I1	R1	C1	M1	A1	N1	T1	**E1**	H1
	G9	P[8]	I1	R1	C1	M1	A1	N1	T1	**E2**	H1

The highest identities between N4006 with RVA G9P[8]-E2 from China and Japan for 10 of the genes suggesting shared ancestors of them. The 100% similarity in some RVA G9P[8]-E2 from China and Japan in NSP3 and NSP5 suggesting shared reassortment ancestors of them with N4006. Significantly, the 100% similarity in RVA G9P[8]-E2 and G2P[4] from Japan and G9P[8]-E2 from China in NSP4 was probably a direct proof that an assortment had happened between China’s recent G9P[8] with Japan’s G2P[4] strain, similar to the previous literature that suggested assortment between vaccine strain and local strain by segment’s 100% nucleotide similarity [40].

In phylogenetic trees, the 11 gene fragments of N4006 had the highest identities and closest genetic relationships with G9P[8]-E2 strains from China in 2019. Except for VP6, all Chinese G9P[8]-E2 strains, including N4006, had the highest identities and closest relationships with Japanese G9P[8]-E2 strains in 2018. Therefore, Chinese G9P[8]-E2 RVA strains, including N4006, had a common ancestor with Japanese G9P[8]-E2 RVA strains. In addition, the NSP4 genes of N4006 and G9P[8]-E2 strains from China and Japan had the highest identities and closest relationships with DS-1-like G2P[4] strains from Japan and Vietnam. Therefore, the NSP4 gene fragments of these G9P[8]-E2 RVAs originated from DS-1-like G2P[4] strains. Interestingly, the reassortment of G3P[8] with the NSP4 gene of DS-1-like RVA occurred in Japan almost simultaneously with that of G9P[8]-E2. This indicated the strong adaptability of Japanese DS-1-like RVA in terms of genetic reassortment with RVAs of prevalent genotypes. Because there is no report of G9P[8]-E2 RVAs in Vietnam and few G2P[4] strains in China at present, Chinese G9P[8]-E2 RVA might originate from an introduction after reassortment between Japanese G9P[8] with Japanese DS-1-like G2P[4] RVAs. In short, Chinese G9P[8]-E2 RVA strains, including N4006, have a common ancestor with Japanese G9P[8]-E2 RVA strains, which might originate from reassortment between Japanese G9P[8] with Japanese DS-1-like G2P[4] RVAs.

Presumed neutralizing epitope analysis of VP7, VP8*, and VP5* showed that N4006 was almost identical to G9P[8] epidemic strains in VP7, including most of recent Chinese RVA strains, and was identical to Wa-like-P[8] strains in VP8* and VP5*. However, it had low identity with vaccines of the same genotype (e.g., ROTASIIL (G9), ROTAVAC (G9), RV1 (P[8]), RV5 (P[8]), and ROTAVIN-M1) and marked differences with vaccines of other genotypes. Altogether, N4006 may have a good protective effect against currently prevalent G9P[8] RVAs and can be used to develop a new rotavirus vaccine. Notably, we successfully isolated and propagated a prevailing RVA epidemic strain to a high titre without introducing adaptation mutations to the virus. Since N4006 represents the dominant epidemic strains in China, and the differences in neutralization epitopes between N4006 and current vaccines suggest the need for vaccine evaluation and the development of a new vaccine. Finding from this study is not without limitation. Although the presumed neutralizing epitopes suggest N4006 is a good candidate vaccine, its actual effectiveness to induce neutralizing mucosal antibodies and protection against current epidemic strains is not tested. This can only be confirmed through animal study in mice model.

## Conclusion

In summary, we adapted the G9P[8] RVA (N4006) in MA104 cells, achieving a high titer (10^5.5^ PFU/mL). The genotype constellation was G9-P[8]-I1-R1-C1-M1-A1-N1-T1-E2-H1 (G9P[8]-E2) and N4006 might originate from reassortment between Japanese G9P[8] with Japanese DS-1-like G2P[4] RVAs. VP7, VP5*, and VP8* of N4006 had low identities with vaccine viruses of the same genotype and marked differences from vaccine strains of other genotypes. The presumed antigenic variation of N4006 with the current vaccine virus warrants evaluation of rotavirus vaccine efficacy against the prevalent RVA G9P[8]-E2 genotype.

## Electronic supplementary material

Below is the link to the electronic supplementary material.

Supplementary Material 1

## Data Availability

All sequences used in this study are available in GenBank. All other relevant information is provided in this current manuscript. If required, the data presented in this work can be shared by e-mail.

## Abbreviations

RVA: Group A rotavirus
DMEM: Dulbecco’s modified Eagle’s medium
CPE: Cytopathic effect
TEM: Transmission electron microscopy
PAGE: Polyacrylamide gel electrophoresis
IFA: Indirect immunofluorescence assay
RVA: Group A rotavirus
WC3: WinStar calf 3
RVGE: Rotavirus gastroenteritis
MOI: Multiplicity of infection
aa: Amino acid

## References

[1] Du Y, Chen C, Zhang X, Yan D, Jiang D, Liu X et al. Global burden and trends of rotavirus infection-associated deaths from 1990 to 2019: an observational trend study. Virol J [Internet]. BioMed Central; 2022;19:1–10. Available from: 10.1186/s12985-022-01898-9. 10.1186/s12985-022-01898-9 PMC9585833 36266651

[2] Tate JE, Burton AH, Boschi-Pinto C, Parashar UD, Agocs M, Serhan F (2016). Global, Regional, and National estimates of Rotavirus Mortality in Children < 5 years of Age, 2000–2013. Clin Infect Dis.

[3] Troeger C, Khalil IA, Rao PC, Cao S, Blacker BF, Ahmed T (2018). Rotavirus Vaccination and the global burden of Rotavirus Diarrhea among children younger than 5 years. JAMA Pediatr.

[4] Steele D, Kirkwood C, Ma L. Next generation rotavirus vaccines WHO Product Development for Vaccines Advisory Committee. 2018.

[5] Mo Z, Mo Y, Li M, Tao J, Yang X, Kong J (2017). Efficacy and safety of a pentavalent live human-bovine reassortant rotavirus vaccine (RV5) in healthy chinese infants: a randomized, double-blind, placebo-controlled trial. Vaccine.

[6] Zhen SS, Li Y, Wang SM, Zhang XJ, Hao ZY, Chen Y (2015). Effectiveness of the live attenuated rotavirus vaccine produced by a domestic manufacturer in China studied using a population-based case-control design. Emerg Microbes Infect.

[7] Zeller M, Patton JT, Heylen E, De Coster S, Ciarlet M, Van Ranst M (2012). Genetic analyses reveal differences in the VP7 and VP4 antigenic epitopes between human rotaviruses circulating in Belgium and rotaviruses in rotarix and RotaTeq. J Clin Microbiol.

[8] Matthijnssens J, Ciarlet M, Heiman E, Arijs I, Delbeke T, McDonald SM (2008). Full genome-based classification of Rotaviruses reveals a common origin between Human Wa-Like and Porcine Rotavirus strains and human DS-1-Like and bovine Rotavirus strains. J Virol.

[9] Fujii Y, Doan YH, Suzuki Y, Nakagomi T, Nakagomi O, Katayama K (2019). Study of complete genome sequences of Rotavirus a epidemics and evolution in Japan in 2012–2014. Front Microbiol.

[10] Li K, Lin XD, Huang KY, Zhang B, Shi M, Guo WP et al. Identification of novel and diverse rotaviruses in rodents and insectivores, and evidence of cross-species transmission into humans. Virology [Internet]. Elsevier; 2016;494:168–77. Available from: 10.1016/j.virol.2016.04.017. 10.1016/j.virol.2016.04.017 PMC7173014 27115729

[11] Sadiq A, Bostan N, Yinda KC, Naseem S, Sattar S (2018). Rotavirus: Genetics, pathogenesis and vaccine advances. Rev Med Virol.

[12] Yu J, Lai S, Geng Q, Ye C, Zhang Z, Zheng Y et al. Prevalence of rotavirus and rapid changes in circulating rotavirus strains among children with acute diarrhea in China, 2009–2015. J Infect [Internet]. Elsevier Ltd; 2019;78:66–74. Available from: 10.1016/j.jinf.2018.07.004. 10.1016/j.jinf.2018.07.004 PMC11373190 30017609

[13] Tian Y, Chughtai AA, Gao Z, Yan H, Chen Y, Liu B (2018). Prevalence and genotypes of group a rotavirus among outpatient children under five years old with diarrhea in Beijing, China, 2011–2016. BMC Infect Dis BMC Infectious Diseases.

[14] Kuang X, Gong X, Zhang X, Pan H, Teng Z (2020). Genetic diversity of group a rotavirus in acute gastroenteritis outpatients in Shanghai from 2017 to 2018. BMC Infect Dis BMC Infectious Diseases.

[15] Duan ZJ, Wang HQ, Diao LD (2021). Expert consensus on immunoprophylaxis of childhood rotavirus gastroenteritis(2020 version). Chin J Appl Clin Pediatr.

[16] Wang M, Ma X, Wei Y, Peng R, Mao T, Wang M (2021). Spread trend analysis on reassortant of rotavirus G9P[8]-E2 in China. Dis Surveill.

[17] Christy C, Madore HP, Pichichero ME, Gala C, Pincus P, Vosefski D (1988). Field trial of rhesus rotavirus vaccine in infants. Pediatr Infect Dis J.

[18] Bucardo F, Rippinger CM, Svensson L, Patton JT (2012). Vaccine-derived NSP2 segment in rotaviruses from vaccinated children with gastroenteritis in Nicaragua. Infect Genet Evol.

[19] Arnold M, Patton JT, Mcdonald SM. Culturing,Storage,and Quantification of Rotaviruses. Curr Protoc Microbiol. 2012;1–29. 10.1002/9780471729259.mc15c03s15 PMC3403738 19885940

[20] Mijatovic-Rustempasic S, Bányai K, Esona MD, Foytich K, Bowen MD, Gentsch JR (2011). Genome sequence based molecular epidemiology of unusual US Rotavirus A G9 strains isolated from Omaha, USA between 1997 and 2000. Infect Genet Evol.

[21] Maes P, Matthijnssens J, Rahman M, Van Ranst M (2009). RotaC: a web-based tool for the complete genome classification of group a rotaviruses. BMC Microbiol.

[22] Phan TG, Okitsu S, Maneekarn N, Ushijima H (2007). Genetic heterogeneity, evolution and recombination in emerging G9 rotaviruses. Infect Genet Evol.

[23] Espínola EE, Amarilla A, Arbiza J, Parra GI (2008). Sequence and phylogenetic analysis of the VP4 gene of human rotaviruses isolated in Paraguay. Arch Virol.

[24] Fujii Y, Oda M, Somura Y, Shinkai T (2020). Molecular characteristics of novel mono-reassortant g9p[8] rotavirus a strains possessing the NSP4 gene of the e2 genotype detected in Tokyo, Japan. Jpn J Infect Dis.

[25] Genggeng K, Hui L, Weiwei L, Yingting Z, Qianwen Y, Hanke W (2021). Gene characterization of human rotavirus Jinzhou strains VP4 and VP7 during 2018 and 2020. Microbiol China.

[26] Settembre EC, Chen JZ, Dormitzer PR, Grigorieff N, Harrison SC. Atomic model of an infectious rotavirus particle. EMBO J [Internet]. Nature Publishing Group; 2011;30:408–16. Available from: 10.1038/emboj.2010.322. 10.1038/emboj.2010.322 PMC3025467 21157433

[27] Venkataram Prasad BV, Chiu W (1994). Structure of rotavirus outer-layer peotein VP7 bound with a neutralizing Fab. Curr Top Microbiol Immunol.

[28] Dormitzer PR, Sun ZYJ, Wagner G, Harrison SC (2002). The rhesus rotavirus VP4 sialic acid binding domain has a galectin fold with a novel carbohydrate binding site. EMBO J.

[29] Morozova OV, Sashina TA, Epifanova NV, Zverev VV, Kashnikov AU, Novikova NA. Phylogenetic comparison of the VP7, VP4, VP6, and NSP4 genes of rotaviruses isolated from children in Nizhny Novgorod, Russia, 2015–2016, with cogent genes of the Rotarix and RotaTeq vaccine strains. Virus Genes [Internet]. Springer US; 2018;54:225–35. Available from: 10.1007/s11262-017-1529-9. 10.1007/s11262-017-1529-9 29236215

[30] Wyatt RG, James WD, Bohl EH, Theil KW, Saif LJ, Kalica AR (1980). Human rotavirus type 2: cultivation in vitro. Sci (80-).

[31] Resch TK, Wang Y, Moon S, Jiang B (2020). Serial passaging of the human Rotavirus CDC-9 strain in Cell Culture leads to attenuation: characterization from in Vitro and in vivo studies. J Virol.

[32] Matthijnssens J, Heylen E, Zeller M, Rahman M, Lemey P, Van Ranst M (2010). Phylodynamic analyses of rotavirus genotypes G9 and G12 underscore their potential for swift global spread. Mol Biol Evol.

[33] Clark HF, Hoshino Y, Bell LM, Groff J, Hess G, Bachman P (1987). Rotavirus isolate WI61 representing a presumptive new human serotype. J Clin Microbiol.

[34] Nakagomi T, Ohshima A, Akatani K, Ikegami N, Katsushima N, Nakagomi O. Isolation and Molecular Characterization of a Serotype 9 Human Rotavirus Strain. Microbiol Immunol [Internet]. 1990;21:99–104. Available from: https://www.unhcr.org/publications/manuals/4d9352319/unhcr-protection-training-manual-european-border-entry-officials-2-legal.html?query=excom 1989. 10.1111/j.1348-0421.1990.tb00994.x 1691433

[35] Urasawa S, Hasegawa A, Urasawa T, Taniguchi K, Wakasugi F, Suzuki H (1992). Antigenic and genetic analyses of human Rotaviruses in Chiang Mai, Thailand: evidence for a close relationship between human and animal rotaviruses. J Infect Dis.

[36] Santos N, Hoshino Y (2001). Global distribution of rotavirus serotypes/ genotypes and its implication for the development and implementation of an effective rotavirus vaccine. Rev Med Virol.

[37] Zhou Y, Li L, Okitsu S, Maneekarn N, Ushijima H (2003). Distribution of human rotaviruses, especially G9 strains, in Japan from 1996 to 2000. Microbiol Immunol.

[38] Khamrin P, Peerakome S, Wongsawasdi L, Tonusin S, Khamrin P, Peerakome S (2006). Emergence of human G9 Rotavirus with an exceptionally high frequency in children admitted to Hospital with Diarrhea in Chiang Mai, Thailand. J Med Virol.

[39] Teodoroff TA, Tsunemitsu H, Okamoto K, Katsuda K, Kohmoto M, Kawashima K (2005). Predominance of porcine rotavirus G9 in japanese piglets with diarrhea: close relationship of their VP7 genes with those of recent human G9 strains. J Clin Microbiol.

[40] Rose TL, da Silva MFM, Goméz MM, Resque HR, Ichihara MYT, de Volotão E (2013). Evidence of vaccine-related reassortment of rotavirus, Brazil, 2008–2010. Emerg Infect Dis.

